# Nature in Disease

**Published:** 1859-04

**Authors:** 


*Nature in Disease;* Illustrated in various Discousres and Essays. To which are added Miscellaneous Writings, chiefly on Medical Subjects. By Jacob Bigelow, M. D., Physician and Lecturer on Clinical Medicine in the Massachusetts General Hospital; Prof. of Materia Medica in Harvard University; President of the American Academy of Arts and Sciences; late President of the Massachusetts Medical Society. Second Edition, enlarged. Boston: Phillips, Sampson & Company, 1859.

By the same author:

*Brief Expositions of Rational Medicine.* To which is prefixed "The Paradise of Doctors," A Fable. P., S. & Co., 1858.

We have just received, from the publishers, the second edition of Dr. Bigelow's interesting little book entitled, "Nature in Disease." The first edition appeared in 1854, and was so well received by the profession as to demand a second edition. The additions which have been made are very few; consisting only of a brief chapter, entitled, "Aphorisms on Cholera," which appeared in the Boston Daily Advertiser in 1832; "The Death of Pliny the Elder," and an article on "The Poisonous Properties of certain American species of Rhus."

Passing in review our sources of information in regard to the death of Pliny the elder, the author is disposed to doubt the accuracy of Mr. Melmoth's translation of that part of the epistle of Pliny the younger, which refers to the death of his uncle. It is generally believed that Pliny died during an eruption of Vesuvius, in the year '79, from suffocation caused by the sulphurous vapors. This supposition is derived from the letter of his nephew who accompanied him in the flight. In refering to the original letter, Dr. Bigelow infers that this was not the cause of death, basing this supposition upon various facts which seem to us most reasonable. He says:—"A medical man may be excused for believing that Pliny died from apoplexy following unusual exertion and excitement, or possibly from a fatal crisis in some disease of the heart previously existing." The volume embraces a number of short papers written in a pleasant and elegant style, and embodying many original and forcible ideas.

In the same style, are the "Expositions of Rational Medicine." This compares the rational mode of practice with the various "pathys" which are now flourishing. To this is prefixed a humorous sketch entitled the "Paradise of Doctors, A Fable," which was read at the annual dinner of the Massachusetts Medical Society. In a style entirely *unique*, it is amusing and contains one or two good "hits" at popular medical prejudices.

